# C-reactive protein: the nexus between inflammation and protein misfolding diseases

**DOI:** 10.3389/fimmu.2025.1612703

**Published:** 2025-06-04

**Authors:** Abhishek Roy, Johannes Zeller, Tracy L. Nero, Johanna Klepetko, Steffen U. Eisenhardt, Michael W. Parker, James D. McFadyen, Karlheinz Peter

**Affiliations:** ^1^ Department of Cardiometabolic Health, The University of Melbourne, Parkville, VIC, Australia; ^2^ Baker Heart and Diabetes Institute, Melbourne, VIC, Australia; ^3^ Department of Plastic and Hand Surgery, Medical Center - University of Freiburg, Faculty of Medicine, University of Freiburg, Freiburg, Germany; ^4^ Department of Biochemistry and Pharmacology, Bio21 Molecular Science and Biotechnology Institute, The University of Melbourne, Parkville, VIC, Australia; ^5^ Australian Cancer Research Foundation (ACRF) Rational Drug Discovery Centre, St. Vincent’s Institute of Medical Research, Fitzroy, VIC, Australia; ^6^ Clinical Laboratory for Bionic Extremity Reconstruction, Department of Plastic, Reconstructive and Aesthetic Surgery, Medical University of Vienna, Vienna, Austria; ^7^ Department of Clinical Haematology, The Alfred Hospital, Melbourne, VIC, Australia; ^8^ The School of Translational Medicine, Monash University, Melbourne, VIC, Australia; ^9^ Department of Cardiology, The Alfred Hospital, Melbourne, VIC, Australia

**Keywords:** C-reactive protein, misfolded proteins, protein aggregation, neurodegenerative diseases, neuroinflammation

## Abstract

C-reactive protein (CRP), an acute-phase protein primarily produced by hepatocytes in response to pro-inflammatory cytokines, is a widely used clinical marker for inflammation and tissue damage. In its native state, CRP exists in a stable pentameric form called pCRP. Upon interaction with activated cell membranes, pCRP undergoes a transitional conformation change into activated pCRP (pCRP*) and subsequently fully dissociates into its monomeric subunits (mCRP). pCRP* and mCRP interact with C1q and thereby activate the classical complement system pathway and both exert pro-inflammatory effects on platelets and endothelial cells. Although classically recognized as a marker of acute inflammation, CRP is increasingly implicated in the pathogenesis of protein-misfolding pathologies, notably neurodegenerative diseases and amyloidosis. This review explores the complex interplay between CRP, encompassing its isoforms pCRP, pCRP*, and mCRP, and misfolded proteins, examining the specific contributions to inflammation and neurodegenerative disease pathogenesis. We analyze the clinical significance of variations in CRP levels in patients with protein-misfolding diseases, discuss underlying mechanisms, and highlight potential implications of these findings for drug discovery and therapeutic targeting of CRP.

## 1 Introduction

Inflammation is increasingly recognized as a key driver of numerous diseases, including chronic conditions such as neurodegenerative disorders (1). C-reactive protein (CRP) is the prototypical acute-phase reactant in humans (2) and a widely used clinical biomarker of inflammation that has traditionally been associated with acute infections and tissue damage (3, 4). It is characterized by its pentameric structure and calcium-dependent ligand binding (5). Despite its early discovery in 1930 (6), its precise biological role remained elusive. Today, CRP is a major focus of research and fast becoming one of the most extensively studied plasma proteins in humans (7). Emerging evidence suggests that CRP is not only an indicator/marker of inflammation, but also a mediator of inflammation playing a complex role in chronic inflammatory states and their associated pathologies (8, 9). This is particularly relevant in the context of neurodegenerative diseases, which are characterized by progressive neuronal dysfunction and often accompanied by chronic inflammation. Neurodegenerative proteinopathies are characterized by the accumulation of misfolded protein aggregates, causing cellular toxicity and contributing to progressive cellular proteostatic collapse (10). Misfolded proteins can be deposited in tissues in the form of amyloid fibrils and cause progressive organ dysfunction.

Interestingly, elevated CRP levels have been observed in patients with various neurodegenerative diseases (11), including Alzheimer’s disease (AD) and Parkinson’s disease (PD) (12). In particular, the onset of AD is strongly associated with higher CRP levels (13). Furthermore, studies have also linked increased CRP levels to an elevated risk of dementia and depression, highlighting the potential impact of inflammation on cognitive decline and mental health (14, 15). In addition to the strong association of elevated CRP levels with neurodegenerative diseases, a recent meta-analysis showed the link between cognitive decline and CRP levels (16). While the exact mechanisms underlying CRP’s involvement in neurodegeneration remain to be fully elucidated, its diverse functions in immune modulation and its interaction with misfolded proteins suggest a multifaceted role in disease pathogenesis. This review aims to provide a comprehensive overview of the current understanding of CRP’s involvement in neurodegenerative diseases and other protein misfolding disorders. We will explore the diverse isoforms of CRP, their respective functions, and their potential contributions to disease progression. Additionally, we will discuss the clinical implications of CRP as a biomarker and its potential as a therapeutic target in these conditions.

## 2 CRP structure and function

**Figure 1:**
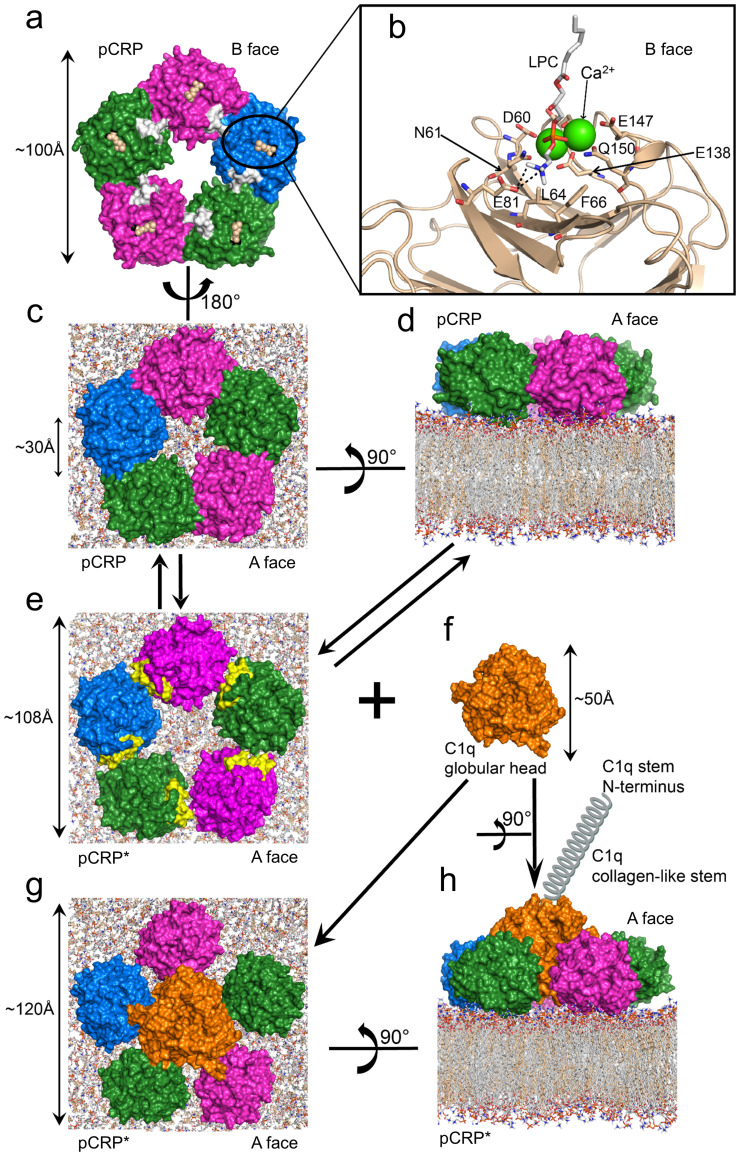
Model demonstrating the conformational change of pCRP to pCRP* and subsequent C1q binding. The native structure of CRP (pCRP) harbors five PCh binding sites located on the binding face (B-face) of the pentamer **(a)**, allowing the PCh headgroups exposed on activated membranes to independently interact with each of the five binding sites **(b–d)**. In **(a)**, the PCh headgroups are shown as cream colored spheres, while in **(b)** lysophosphatidylcholine (LPC) is depicted as white/blue/red/orange colored sticks. The detection of neoepitopes **(e)**, (residues 199-206, yellow), using anti-mCRP/pCRP* antibodies 9C9 and 3H12, indicates that the native pentamer has begun to dissociate to pCRP*. Further dissociation of the CRP pentamer enables binding of the globular head from a single chain in the C1q hexamer **(f–h)**. Each chain in the C1q hexamer is comprised of a collagen-like stem (gray spring), **(h)** connecting the C-terminal globular head (orange) to the N-terminal stalk (not shown). When at least three chains in a C1q molecule interact with pCRP* in a 1:1 ratio, activation of the complement C1 complex occurs. The initial conversion of pCRP to pCRP* appears to be a reversible process. The crystal structures of the PCh-pCRP complex (PDB ID: 1B09 (17)) and the C1q globular head (PDB ID: 1PK6 (18)) were used to construct the models, one letter amino acid codes are shown in **(b)**. Reproduced from Braig et al. (19) in accordance with the Creative Commons CC BY license.

The native and relatively inert form of CRP is composed of five identical subunits, each ~23 kDa in size, and the resulting pentamer has an overall donut-like or disc-like shape (**Figures 1a–d**). Non-covalent interactions (electrostatic and hydrophobic interactions) at the subunit-subunit interfaces hold the pentamer together. pCRP is primarily synthesized in hepatocytes under the regulation of interleukin-6 (IL-6), and initially each of the five subunits folds into an approximately native monomeric core (20, 21). Next, an intrachain disulfide bond is formed between Cys36 and Cys97 and the binding of two calcium ions completes the monomeric subunit structure. Each monomeric subunit is comprised of one α-helix (residues 168-176) and two approximately parallel β-sheets, each containing seven β-strands. Subsequently, five subunits assemble into the native pCRP. The calcium ions are held into place largely by acidic residues (Asp60, Glu138, Asp140 and Glu147), and also Asn61 and Gln150, located at one end of a β-sheet (**Figure 1b**). The calcium ions are crucial for subunit folding and the subsequent pentamerization (19, 22, 23). Indeed, mutations in the pCRP calcium binding site prevents proper assembly and release of CRP from transfected cells (24).

The donut-shaped native pCRP has two exposed faces, these are called the effector face (also known as the “A-face” or activating face) and the binding face (or “B-face”), respectively (**Figure 1**). The effector face is responsible for triggering the innate immune pathway by binding ligands such as Fcγ receptors and the complement C1 complex. The binding face adheres to damaged or apoptotic cell membranes, bacterial cell walls and has also been reported to bind to β-sheets. On the binding face of pCRP, each monomeric subunit contains a shallow groove where the phosphocholine (PCh) and phosphoethanolamine headgroups of bioactive lipids bind in a calcium-dependent manner. These lipid headgroups are exposed on damaged or inflamed cell membranes (17) (**Figure 1**). Site-directed mutagenesis and co-crystallization studies targeting the PCh binding sites of pCRP have indicated that Phe66 and Glu81 are important for the interaction with the lipid headgroups (25, 26). In each subunit, Phe66 is located on the edge of the shallow groove approximately halfway between Glu81 and the two calcium ions, and it interacts with the amine methyl groups of PCh (**Figure 1b**). The PCh (and similarly phosphoethanolamine) headgroup is anchored to the binding site (i.e., shallow groove, **Figure 1b**) by two critical interactions: (1) the coordination of the negatively charged PCh phosphate moiety with the positively charged calcium ions and (2) the interaction of the positively charged PCh amine nitrogen with the negatively charged acidic side-chain of Glu81. Residues Phe66 and Glu81 are highly conserved across all species, underscoring their importance in pCRP binding to bioactive lipid headgroups such as PCh and phosphoethanolamine (27).

The pentameric structural state of CRP is dependent upon its environment (19). Under normal physiological conditions it adopts the native non-inflammatory pentameric state (pCRP, **Figures 1a–d**), whereas in localized inflammatory environments it adopts a non-native activated pentameric state (pCRP*, **Figures 1e, g, h**) (19, 28, 29). The trigger for adopting the pro-inflammatory activated pCRP* state occurs when pCRP binds to PCh (or phosphoethanolamine) headgroups exposed on the surface of damaged cells (19). The exposed lipid headgroups can access the PCh binding site on each subunit in the binding face (B-face, **Figures 1c, d**) of pCRP, in addition the headgroups can interact with residues at the subunit-subunit interface thereby weakening the interactions holding the CRP pentamer together. The increase in both curvature and fluid nature of the lipid bilayer in damaged cell surfaces exposes the PCh (or phosphoethanolamine) headgroups further and the resulting mechanical shear force pulls the CRP subunits apart (19, 30). Detection of the native pCRP isoform can be achieved using antibodies which recognize the circulating pentamer, such as anti-pCRP-8D8 (19). The dissociation of pCRP can be detected using anti-mCRP/pCRP* antibodies, such as 9C9 and 3H12 (19, 31, 32). These two anti-mCRP/pCRP* antibodies target residues 199–206 within a CRP subunit and this region is often referred to as the neoepitope. The neoepitope is normally buried at the subunit-subunit interface in pCRP and not accessible for antibody binding. When the pCRP subunits start to dissociate (**Figure 1e**), the neoepitopes are exposed while an overall pentameric structure (pCRP*) is retained (19, 30). Once the distance between adjacent subunits in pCRP* is > 4 Å (**Figures 1g, h**), the subunits are only loosely associated with each other and there are three main outcomes for pCRP*. Data obtained by us, and others, indicates that the transformation from pCRP to pCRP* is somewhat reversible, therefore one outcome is that the subunits move back together and pCRP is reformed (19). A second outcome is that the pCRP* subunits continue to move apart, and at a distance of ~10 Å the dissociation process becomes irreversible. At this point complete dissociation of the pentamer occurs, the disulfide bond between Cys36-Cys97 is now exposed and can be reduced. Breaking of the disulfide bond leads directly to the individual CRP subunits unfolding, and results in the production of the pro-inflammatory monomeric form of CRP (mCRP) which is subsequently cleared from circulation (20). Interestingly, the conformational change from pCRP (largely β-sheet secondary structure) to mCRP (largely disordered secondary structure) leads to a significant lowering in the protein’s solubility, transforming from a soluble pentamer to an insoluble, tissue-bound monomer aggregate (29–31).

The third outcome for pCRP* is the interaction with an effector ligand such as the complement C1 complex, to activate the classical complement pathway. The C1 complex is comprised of components C1q, C1r and C1s. C1q, a hexameric protein, is known to directly interact with CRP (23). Each chain in the C1q hexamer is comprised of an N-terminal stalk connected to a collagen-like stem (or arm) with a C-terminal globular head (**Figures 1f–h**) (18, 19, 23). The collagen-like stems splay out such that the C1q hexamer resembles an upside down bunch of flowers. The main interaction of C1q with CRP, determined by mutagenesis, is via residues located in the C1q globular head. CRP residues identified by mutagenesis to be involved in binding the globular head of C1q are located on the pCRP effector face (A-face) toward the pentameric ring interior (18, 23, 33–35). We utilized the crystal structure of the C1q globular head (PDB ID: 1PK6 (18)) to model the interaction with CRP (**Figures 1f–h**) (19). The diameter of the globular head of C1q is too large for it to interact with the CRP residues located on the interior surface of the native pCRP pentamer ring, and we proposed that the C1q globular head instead interacts with pCRP*. The model for the interaction of one pCRP* molecule with one C1q globular headgroup is shown in **Figure 1** and is consistent with our published data demonstrating that C1q is recruited by microvesicle-bound pCRP* (19). The model is also consistent with a 24 Å low-resolution cryo-electron tomography structure for the CRP-C1 complex published in 2024 by Noone et al. (23). Since C1q is a hexamer, there is the potential for all six C-terminal globular heads to independently bind to six pCRP* molecules. Although their CRP-C1 complex structure is low-resolution, Noone et al. were able to demonstrate that four C1q chains interact (via their globular headgroup) simultaneously with four pentameric CRP molecules laid out in a raft-like configuration bound to the surface of a liposome, with two C1q chains remaining free. It may be that the curvature of the liposome surface prevents all six C1q chains from binding to six pentameric CRP molecules simultaneously, however this remains to be explored using liposomes of varying diameters. While Noone et al. proposed that the C1q globular headgroups are interacting with pCRP, the observed density would also accommodate pCRP* and the authors acknowledge that the diameter of the globular head of C1q is too large to interact with the interior surface of the native pCRP pentamer ring (23). Clarification of the precise nature of the pentameric form of CRP observed by Noone et al. will require higher resolution cryo-electron tomography or cryo-electron microscopy data (23).

The idea that non-inflammatory native pCRP undergoes structural modulation, to pCRP* and mCRP (and possibly other as yet undetermined isoforms), at sites of tissue damage to exhibit its pro-inflammatory response has also been proposed by others (36, 37). As described above, the canonical activation mechanism of CRP includes the protein’s interaction with PCh headgroups exposed on activated cell membranes. These activated cells typically have increased levels of phospholipase A2 activity, a key enzyme in the production of the lipid lysophosphatidylcholine (LPC, **Figure 1b**) (31). Several studies, including our own research findings, have demonstrated that pCRP can undergo complete dissociation into the pro-inflammatory monomeric form of CRP (i.e., mCRP) on the surface of endothelial cells (31), activated platelets (38), monocytes (19) and microparticles (39), which are all rich in lipids containing PCh headgroups (40). In addition to the PCh-dependent dissociation mechanism described above, pCRP can also dissociate via PCh-independent mechanisms. For example, we recently described a novel mechanism of PCh-independent, shear-induced pCRP dissociation relevant to pathologies involving increased shear rates, such as aortic stenosis, atherosclerotic and injured arteries (30).

As discussed above, dissociation of pCRP to the pCRP* isoform enables the binding of C1q within the C1 complex, and the subsequent activation of the classical complement pathway (19). pCRP* located on the surface of microparticles secreted from activated cells contributes to tissue inflammation. In our previous work, we found that pCRP* constitutes a substantial percentage of all CRP isoforms present in injured tissues including burn wounds, atherosclerotic plaques and inflamed muscle (19). Due to its low solubility and exceedingly disordered structure, mCRP is likely to be removed quickly *in vivo*, whereas microparticles can act as a chaperone for pCRP* (41). This then raises the question: is mCRP the major pro-inflammatory isoform of CRP or can this activity be largely attributed to pCRP*? The surge in research into characterizing CRP isoforms and their specific biological functions will no doubt provide the answer to this question and may facilitate new treatment opportunities for inflammatory diseases.

## 3 Protein misfolding diseases

### 3.1 Protein folding

In addition to the pro-inflammatory structurally disordered mCRP being formed via the PCh-dependent dissociation of pCRP, as detailed above, we have reported the generation of mCRP through the direct interaction of non-inflammatory pCRP with misfolded proteins (42). Furthermore, co-localization of both C1q and CRP with amyloid plaques (insoluble clumps of tangled amyloid fibrillar proteins) suggests the involvement of pCRP* (and potentially mCRP) and implies that CRP interactions with misfolded proteins play a central role in inducing immune responses (42). Misfolded proteins are thermodynamically unstable and tend to interact with diverse macromolecules, including functional proteins, forming complex interactomes that can drive disease pathogenesis (43). In contrast, correctly folded polypeptides achieve unique, thermodynamically stable, functional, three-dimensional native conformations during their synthesis.

**Figure 2:**
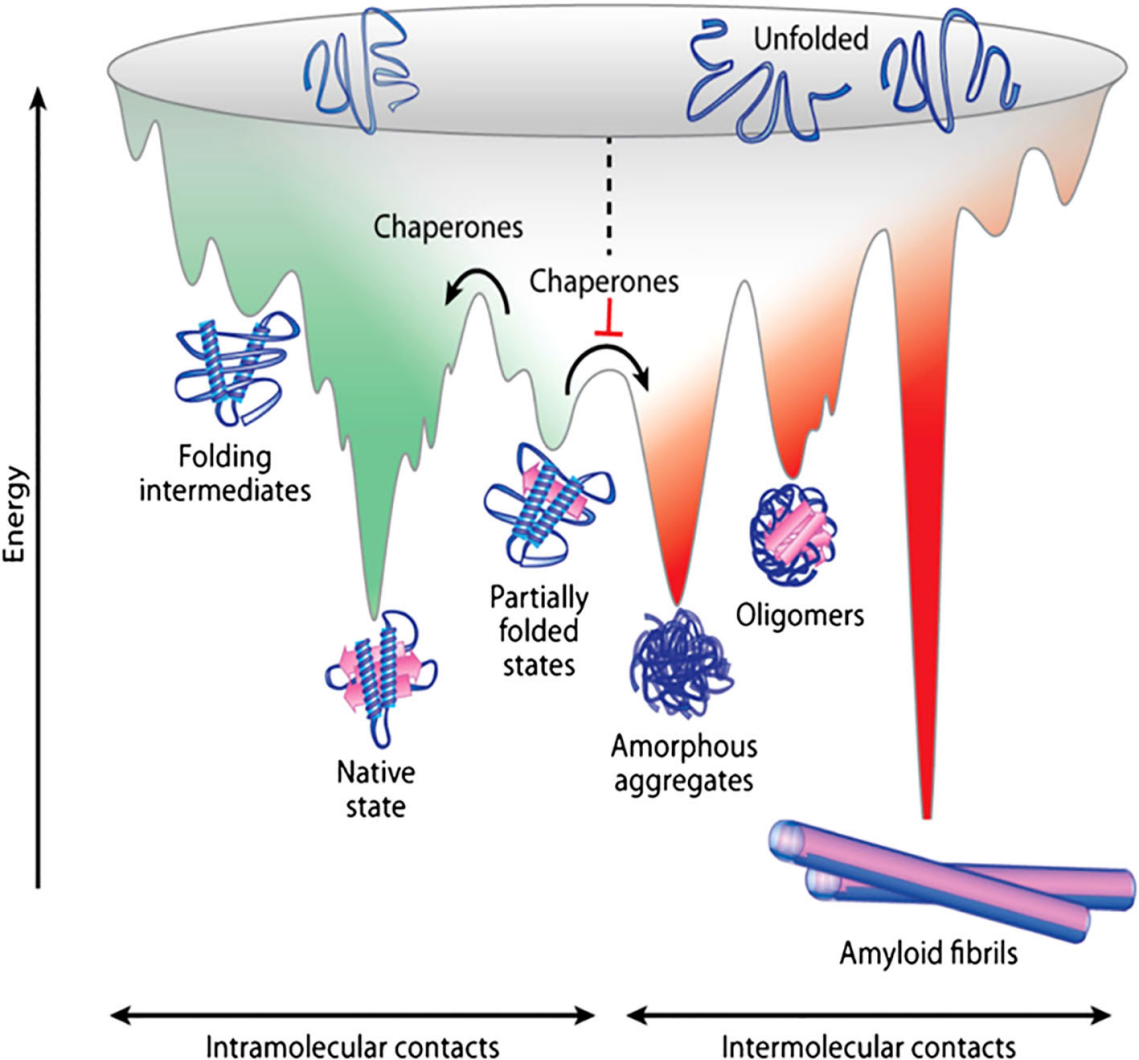
The protein folding and aggregation energy landscape. Schematic diagram depicting the energy landscape guiding protein folding. Newly synthesized polypeptides navigate a multitude of possible conformations to achieve their unique, functional three-dimensional structure. The green surface represents the folding funnel, where intramolecular contacts drive the unfolded protein toward its native state. The red surface illustrates pathways leading to protein aggregation, either amorphous aggregates or amyloid fibrils, driven by intermolecular interactions. The overlap between the green and red regions highlights the competition between folding, unfolding and aggregation. Molecular chaperones (e.g., Hsp70 (44) or microvesicles (19) etc.) typically prevent protein aggregation. Aggregation can arise from intermediate conformations encountered during *de novo* protein folding or from the destabilization of the native state into partially unfolded conformations. Importantly, cell-toxic oligomers can form as off-pathway intermediates during amyloid fibril formation. Figure reproduced from Muntau et al. (45) with permission.

Rather than following a single pathway, protein folding typically proceeds through a number of self-modulating processes guided by the folding funnel energy landscape (**Figure 2**) (45). This landscape depicts the energy of various conformational states for a protein, each state decreasing in energy as the protein progresses toward a more organized, native-like structure. At the initial folding stage, secondary structure elements (e.g., α-helices and β-strands) form at specific locations along the polypeptide chain, coinciding with a decrease in free energy of the system. As the protein approaches its precise native folded state, characterized by a unique arrangement of the secondary structure features, it reaches its lowest free energy, achieving both structural and functional stability (46). This efficient and rapid search for the native state is often visualized as a funnel, where the unfolded protein occupies a high entropy and high free energy state representing a multitude of random conformations (**Figure 2**). The narrowing of the funnel reflects a progressive reduction in the number of accessible protein conformations and concomitant decreases in free energy and entropy (47). Chaperone-assisted folding of proteins achieves relatively lower energy minima (**Figure 2**), however, in the absence of chaperones, proteins may misfold or aggregate. Under certain conditions, misfolding or aggregation may lead to the protein adopting an amyloid state, which is often the lowest energy minimum conformation (**Figure 2**). While the funnel analogy typically depicts one of the lower energy minima as the native protein conformation, a protein can possess an ensemble of native or near-native conformations, all crucial for its biological function. The rapid and efficient navigation of the folding landscape is facilitated by a network of interactions between key residues, often forming a folding nucleus that establishes the native topology within the transition state ensemble (the folding bottleneck) (6). The network of key interactions effectively guides the protein through the complex energy landscape to its native, functional state.

### 3.2 Protein misfolding and aggregation

Protein folding generally involves several transitional conformation states and may encounter rate-limiting steps. While most transition states lead to functional folding of proteins, some can result in non-native interactions, promoting protein aggregation (48). There is usually a significant energy barrier between the native protein state and aggregated states (**Figure 2**). Native protein folded states are often metastable, and kinetic partitioning plays a crucial role in preventing misfolding and aggregation (49). Kinetic partitioning is influenced by both extrinsic and intrinsic factors. Extrinsic factors include quality control machinery, such as molecular chaperones, which assist in native functional folding, suppress misfolding, and target misfolded proteins for degradation (50). The heat shock protein 70 kDa (Hsp70) family of chaperones, for example, prevents misfolding and aggregation under stress. Chaperone-mediated autophagy also contributes to the degradation of misfolded and aggregated proteins (51). For instance, Hsp70 and Parkinsonism-associated deglycase facilitate the autophagic degradation of unstable neuronal proteins that are the substrates for aggregation and α-synuclein (α-syn) aggregates, respectively (52, 53). Control of translation dynamics, through fluctuations in tRNA abundance and tuning of tRNA-ribosome binding affinity, represents another extrinsic regulatory mechanism influencing kinetic partitioning (54, 55).

Intrinsic factors are shaped by evolutionary pressure, leading to the selection of so called “aggregation gatekeepers” that enhance the kinetic partitioning. These gatekeepers contain specific sets of residues, such as proline (which disrupts β-sheets) or electrostatically charged residues, e.g., lysine, arginine, aspartate, or glutamate, that neutralize aggregation-prone interactions (56). Located within the aggregation prone regions (APRs), these gatekeepers effectively reduce aggregation propensity (57). Loss-of-function, dysfunction or absence of aggregation gatekeepers is frequently observed in intrinsically disordered proteins (IDP) and intrinsic disordered protein regions (IDPR) (58), and contributes to amyloid-related diseases. Examples include α-syn in PD, tau and amyloid beta (Aβ) in AD, TAR DNA-binding protein 43 in amyotrophic lateral sclerosis (ALS), Huntingtin protein in Huntington’s disease (HD), and transthyretin (TTR) in systemic amyloidosis (59, 60). IDP/IDPR, due to their conformational flexibility and rugged energy landscapes, are particularly susceptible to intermolecular interactions that can lead to the formation of oligomers and amyloid fibrils (61).

### 3.3 Protein aggregates: oligomers and fibrils

Protein aggregates, encompassing both oligomers and fibrils, play a central role in neurodegenerative and systemic amyloidosis. While amyloid fibril deposition, both extra- and intracellularly, is a hallmark of these diseases, accumulating evidence points to the toxic nature of soluble oligomers (62, 63). Oligomers, characterized by high hydrophobicity and a large surface-to-volume ratio (64), can disrupt subcellular functions, including membrane integrity, metal ion homeostasis, redox balance, and endolysosomal pathways (65–67). Furthermore, oligomers stall subcellular activities and directly interfere with the function of other proteins. For example, α-syn oligomers impair mitochondrial respiration by inducing depolarization and interacting with the respiratory chain complex I (68). Oligomeric α-syn also interacts with Tom20, a mitochondrial outer membrane protein, hindering mitochondrial protein import (69). Similarly, tau and Aβ oligomers disrupt mitochondrial function by impairing the respiratory chain complex activity (70, 71). Calcium dyshomeostasis and membrane disruption are common consequences of oligomer toxicity (72). Oligomers also contribute to elevated reactive oxygen species (ROS) levels. Intriguingly, β-sheet-rich oligomers have been shown to induce significantly higher ROS production in astrocyte-neuron co-cultures compared to unstructured oligomers and monomers (73, 74). While intracellular aggregates are typically cleared by endolysosomal pathways, these pathways can be disrupted by oligomers. For instance, phagocytosis of α-syn oligomers stall the endolysosomal pathway resulting in an exponential accumulation of α-syn aggregates (75). Tau oligomers, although also targeted for clearance via the endolysosomal pathway, can themselves obstruct this pathway, leading to lysosomal dysfunction (76). The impairment of phagocytosis contributes to increased aggregation kinetics and the formation of stable fibrils. Amyloid fibrils, insoluble and highly ordered aggregates, are implicated in various diseases, including neurodegenerative disorders like AD, PD, HD, ALS, and prion diseases (77–79). Beyond neurodegenerative diseases, amyloidosis can affect other tissues and organs, such as the pancreas (islet amyloid polypeptide), the heart (light chain amyloids and transthyretin amyloids) and kidneys (light chain amyloids) (80). In systemic amyloidosis, the transfer of amyloid fragments, termed “proteopathic-seeds”, between tissues can initiate fibrillation in recipient tissues (81–83). While the pathological relevance of this seeding phenomenon requires further investigation for many proteins, it is well-established in prion diseases (84).

The formation of misfolded protein oligomers and fibrils is widely recognized as a primary driver of immune activation, making inflammation a central component of neurodegenerative disease. Both stable, large fibrillar aggregates (fibrils) and unstable, smaller oligomeric assemblies (oligomers) can initiate immune cell activation, thereby triggering inflammatory signaling cascades e.g., nuclear factor-κB signaling (85, 86). This activation results in the elevated expression of pro-inflammatory cytokines (including IL-1β and tumor necrosis factor α (TNFα)), as well as CRP, which collectively contribute to exacerbated cellular dysfunction and cell death (87).

## 4 CRP and neurodegenerative diseases

Neurodegenerative diseases represent a diverse group of neurological disorders characterized by the progressive loss of neurons in the peripheral nervous system (PNS) and/or central nervous system (CNS), affecting millions globally (88). The neuronal loss results in structural disintegration and dysfunction of neural networks, which, due to the terminally differentiated nature of neurons, exhibit limited regenerative capacity (88). Beyond neuronal loss, progressive localized inflammation is a hallmark of neurodegenerative diseases (89). While initially attributed to protein aggregation, emerging evidence suggests that inflammation is relevant in the early phase of the disease process, potentially triggered by various pro-inflammatory macromolecules within the plaque microenvironment (90). The immune system, crucial for maintaining tissue homeostasis by clearing inflammatory stimuli, such as protein aggregates, can become dysregulated in neurodegenerative diseases. Failure to effectively resolve inflammation leads to chronic activation and secretion of neurotoxic factors, exacerbating disease progression (91). Consequently, vital communicative circuits are disrupted, resulting in impairments across sensory, motor, cognitive and behavioral domains (1, 92).

CRP is considered a key inflammatory marker and a promoting player in acute inflammation, suggesting it may have a significant role in neurodegenerative inflammatory processes (11, 93). Though the precise mechanisms by which CRP interacts with immune cells and pro-inflammatory molecules are still not clearly understood, its involvement in neurodegenerative disease progression is well documented (11, 37). Elevated serum CRP levels are consistently observed in PD patients, with levels correlating to disease severity as assessed by the Hoehn-Yahr staging system (94). Similarly, patients with ALS exhibit significantly higher CRP levels compared to healthy controls (95). Furthermore, the presence of CRP within senile plaques in AD suggests its potential contribution to the acute inflammatory response associated with plaque formation (96).

### 4.1 CRP and Alzheimer’s disease

Alzheimer’s disease (AD) is characterized histopathologically by the accumulation of extracellular Aβ peptides, phosphorylated tau (p-tau), and the formation of senile plaques. Aβ peptides are generated through the proteolysis of amyloid precursor protein (APP) by α-, β-, and γ-secretases (97). Cleavage by α- and β-secretases produces soluble APP fragments, (sAPPα and sAPPβ, respectively), which can be further processed by γ-secretase to release Aβ peptides, P3 peptides, and the APP intracellular domain (AICD) (98). Importantly, secretase activity can be modulated by inflammatory cytokines, suggesting a potential link between inflammation and Aβ production (99). This supports the hypothesis that individuals with pre-existing inflammatory conditions may be more susceptible to AD pathology (100, 101).

Neuroinflammation is a key contributor to AD pathogenesis. Initial inflammatory stimuli, such as Aβ deposition, pathogenic infection or cellular debris, activate microglia, leading to the release of pro-inflammatory cytokines (IL-6, IL-1β, TNFα) and chemokines (CCL)-2, CCL-4 and CCL-11 (102, 103). This process, while typically tightly regulated, can become dysregulated in AD. Excessive Aβ and p-tau accumulation overwhelms the microglial clearance capacity, resulting in sustained inflammation and the recruitment of reactive astrocytes. These astrocytes and microglia contribute to a chronic inflammatory environment, further exacerbated by the release of damage-associated molecular patterns (DAMPs) from necrotic cellular debris (104). The persistent inflammation contributes to neuronal dysfunction and ultimately neurodegeneration.

The association between CRP levels and AD is influenced by various factors, including gender, age, socioeconomic status, and pre-existing morbidities. For example, Schmidt et al. found that elevated CRP levels were associated with a threefold higher risk of dementia in men and that even moderately increased CRP levels were linked to later-life AD development (105). More recent studies have employed Mendelian randomization to investigate the causal relationship between CRP and AD. Zhang et al. utilized genome-wide meta-analysis data from 383,378 controls and 71,880 AD patients, identifying 56 single nucleotide polymorphisms (SNPs) associated with CRP levels (13). Their analysis revealed a significant association between genetically predicted elevated CRP levels and increased AD risk. These findings are consistent with previous studies demonstrating a link between AD and elevated CRP, along with other pro-inflammatory markers. Moreover, high CRP levels are associated with elevated serum cholesterol, a known risk factor for AD (13). Sensitivity analyses showed similar results, and no pleiotropic bias was observed. Several studies have shown a significant link between AD and CRP, along with higher levels of pro-inflammatory markers including IL-1β, IL-6, and α-1-antichymotrypsin (106).

**Figure 3:**
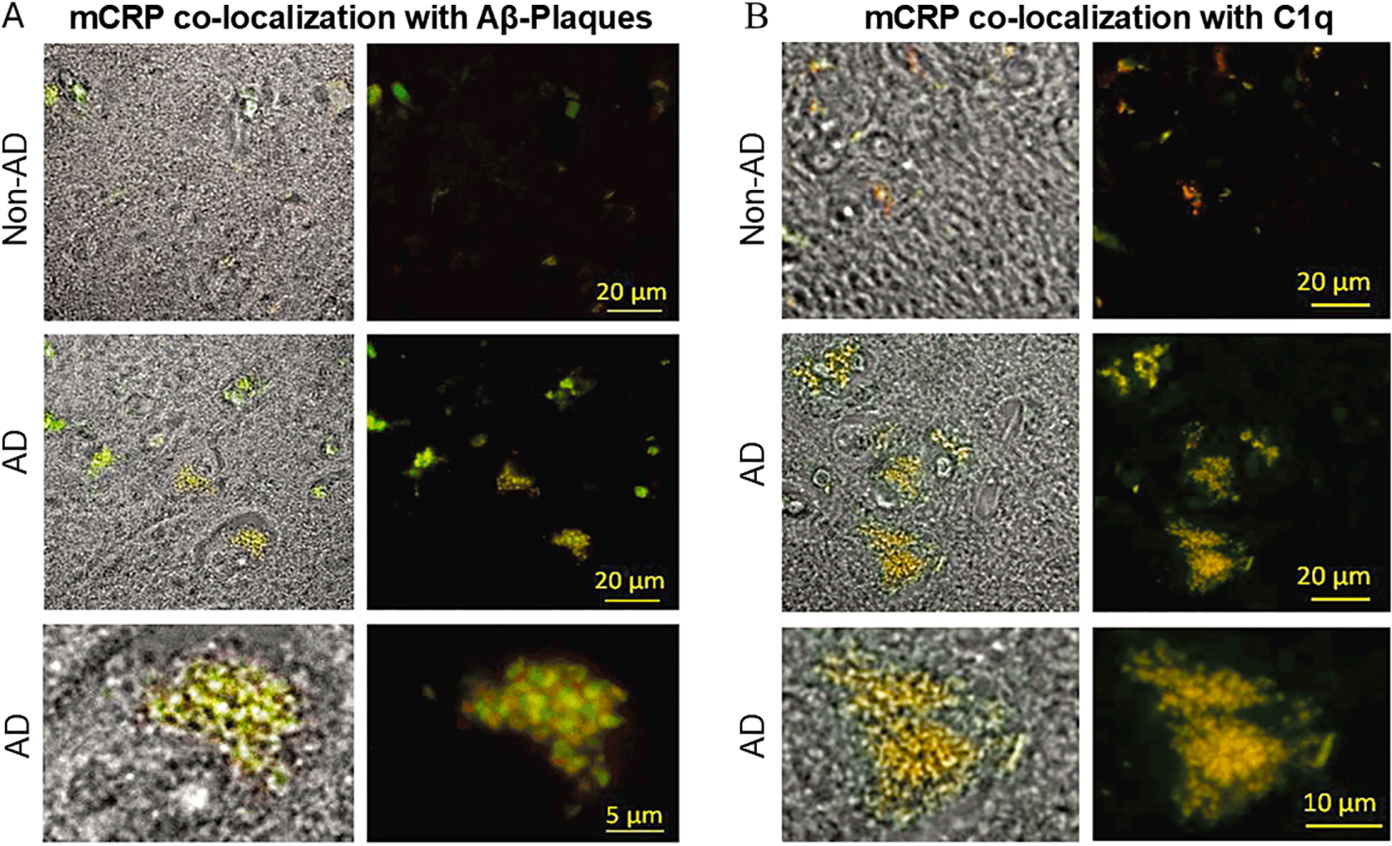
Identification of pCRP* and/or mCRP as the predominant CRP isoforms in human brain tissue from AD patients and co-localization with C1q and Aβ plaques. **(A)** Bright field and fluorescence microscopy images demonstrating co-localization of pCRP* and/or mCRP with Aβ plaques in AD brain tissue. pCRP* and/or mCRP (red) and Aβ plaques (green) signals overlapped in AD tissue, indicating co-localization. **(B)** Co-localization of pCRP* and/or mCRP with C1q in AD brain tissue. pCRP* and/or mCRP (red) and C1q (green) signals overlapped in AD tissue, indicating co-localization. Healthy control tissue showed minimal staining for pCRP* and/or mCRP and C1q; reproduced from Strang et al. with permission (83).

In addition to a causal role in AD, CRP appears to play a central role in eliciting inflammation in this disease. As discussed above, CRP activates the classical complement system, leading to cell lysis and phagocytosis (107). In the context of AD, complement activation can contribute to neuronal damage and the lysis of healthy cells. Complement proteins are produced in the CNS by astrocytes, microglia and neurons and levels of these proteins are elevated in AD, PD and other neurodegenerative diseases. It has been demonstrated that the C1q component of the complement C1 complex can bind directly to Aβ fibrils in the brains of AD patients, thereby activating the classical complement pathway (108–111). Notably, complement pathway inducers, including the pro-inflammatory CRP isoforms pCRP*/mCRP, have also been found in AD lesions (112). Our research work further elucidated the role of CRP in AD by demonstrating that Aβ plaques can induce the dissociation of native pCRP into its monomeric form (i.e., mCRP) via the pCRP* isoform. In work published in 2012, we observed co-localization of mCRP (using 9C9 antibody) with Aβ plaques (using NAB228 antibody) in AD patients, but no significant difference in pCRP staining between AD patients and normal controls. Additionally, co-localization of C1q and mCRP was observed in AD patients (**Figure 3**). By 2017 we, and others, had identified a pro-inflammatory pentameric form of CRP, which we dubbed pCRP* (20). This pro-inflammatory CRP isoform is also recognized by antibody 9C9, necessitating a reinterpretation of our 2012 data, i.e., that both pCRP* and mCRP could be co-localized with Aβ plaques. These findings suggest that Aβ possesses an intrinsic ability to convert pCRP to pCRP* and/or mCRP, thereby amplifying neuroinflammation (42, 113–115). The interaction of C1q with Aβ fibrils is mediated via the C1q globular headgroups (108–110), the same region of C1q involved in the interaction with pCRP* (**Figures 1g, h**). Thus, it is likely that upon binding to Aβ fibrils, pCRP dissociates to pCRP* followed by recruitment of C1q globular headgroups and the subsequent activation of the C1 complex. This process would enable a direct interaction of the C1q globular head with both the surface of the Aβ fibril and the CRP residues lining the inner ring surface of pCRP*. The scenario can be visualized by replacing the model cell membrane in **Figure 1** with an atomic-level structure of an Aβ fibril. It is now possible to investigate the existence of an Aβ fibril:pCRP*:C1q complex using cutting edge cryo-electron microscopy and cryo-electron tomography techniques. Furthermore, Gan et al. demonstrated that mCRP can induce p-tau and Aβ42 expression in primary neurons in an apolipoprotein ϵ (APOE) genotype-dependent manner (116). Their study revealed that mCRP differentially affects APP and β-site APP cleaving enzyme-1 expression levels and Aβ production in neurons expressing different APOE isoforms, highlighting the complex interplay between CRP, APOE genotype, and AD pathology. Taken together, these findings strongly implicate CRP as a key mediator of neuroinflammation and a potential therapeutic target in AD (116).

Not only do pCRP-Aβ interactions amplify neuroinflammation, pCRP has emerged as a potential key modulator of Aβ aggregation (115). For example, Ozawa et al. deciphered the anti-amyloidogenic property of pCRP as being attributable to pCRP directly inhibiting aggregation of Aβ(1-40) (113). Whereas Ngwa et al. showed that Aβ(1-42) aggregation was unaltered in the presence of native pCRP but inhibited when in the presence of a mutant pCRP. Mutation in the PCh-binding site on the B-face of pCRP resulted in significant inhibition of Aβ(1-42) aggregation (114). However, these studies did not consider the formation of pCRP* and/or mCRP while incubating Aβ-pCRP together, and these CRP isoforms could be the anti-amyloidogenic agent(s) rather than native pCRP.

### 4.2 CRP and Parkinson’s disease

Parkinson’s disease (PD), a common neurodegenerative disorder, is characterized by motor symptoms including rigidity, bradykinesia, postural instability, tremor, and gait abnormalities (117). Neuropathologically, PD is defined by a significant reduction in dopamine levels within the substantia nigra pars compacta, resulting from the selective degeneration of dopaminergic neurons, and the presence of intraneuronal inclusions known as Lewy bodies and Lewy neurites (118). These inclusions are primarily composed of aggregated α-syn, which disrupts lysosomal and mitochondrial function (119). Both wild-type and mutant α-syn are prone to aggregate; however, the mutant forms exhibit a higher propensity for oligomer and fibril formation, indicating a central role for α-syn dysfunction in familial PD. The presence of pathological α-syn oligomers and fibrils at synapses suggests that early synaptic dysfunction is a key initiating event in PD pathogenesis (120, 121). While α-syn aggregates are predominantly intracellular, oligomers can also be found in cerebrospinal fluid and plasma, potentially seeding the fibrillation process in neighboring neurons. Lewy body formation represents a later stage, wherein α-syn oligomers coalesce into pale bodies within the neuronal cytoplasm (122). This seeding process induces the formation of stable fibrils from smaller α-syn aggregates, ultimately forming classic Lewy bodies. Although Lewy bodies contribute to neuronal dysfunction by occupying cytoplasmic space, evidence suggests that α-syn oligomers, rather than fibrils, may be the primary drivers of cellular homeostasis disruption in PD (123).

The mechanisms underlying immune activation in PD remain complex. α-syn aggregates trigger innate immune responses by interacting with Toll-like receptors (TLRs) on peripheral monocytes and microglia, leading to T-cell infiltration into the brain and subsequent adaptive T-cell responses (124). In PD, CD4+ and CD8+ T-cells are enriched in the brain parenchyma, correlating with neuronal damage. Mitochondrial dysfunction, a hallmark of PD, can also initiate immune activation. Major histocompatibility complex (MHC) class I, a mitochondrial antigen, can bind to CD8+ T-cells and upregulate cytokine expression (125). α-syn-mediated T-cell responses are linked to human leukocyte antigen (HLA) alleles, with α-syn aggregates interacting with HLA variants, suggesting a genetic basis for HLA association with PD risk (126, 127). Upon T-cell receptor (TCR) binding to MHC-presented antigen, CD4+ T-cells release effector cytokines, stimulating B-cells and CD8+ T-cells, which in turn secrete pro-inflammatory cytokines such as interferon-γ, IL-2, and TNFα (128). These cytokines can upregulate CRP expression, potentially exacerbating neuroinflammation and contributing to neurodegeneration. Consistent with this, meta-analyses have shown elevated CRP levels in both cerebrospinal fluid and peripheral blood of PD patients compared to healthy controls (16). Studies have further elucidated the association between CRP and PD pathology. Moghaddam et al. demonstrated that CRP concentrations in cerebrospinal fluid correlate with both motor and non-motor symptoms, suggesting a role for neuroinflammation in the onset as well as progression of PD (12). Choi et al. found an inverse correlation between serum CRP levels and cognitive function, as assessed by Mini-Mental State Examination (MMSE) scores, indicating a potential link between increased CRP and cognitive decline in PD (129). Furthermore, baseline CRP levels have been associated with increased mortality risk in PD, independent of cognitive function, disease severity, and duration, highlighting the potential involvement of neuroinflammation in neurodegenerative pathways (130). Similarly, Sawada et al. reported a logarithmic-linear association between baseline CRP levels and mortality risk, with a shorter mean survival time observed in PD patients with higher CRP levels (98). Additionally, studies have shown that baseline CRP levels correlate with motor deterioration and prognosis, independent of other clinical factors (131). These findings suggest that subclinical systemic inflammation, as reflected by elevated CRP, may contribute to and exacerbate neurodegeneration in PD.

### 4.3 CRP and amyotrophic lateral sclerosis

Amyotrophic lateral sclerosis (ALS) is a devastating neurodegenerative disease characterized by the progressive degeneration of both upper and lower motor neurons, originating in the brainstem nuclei, spinal cord ventral roots, and motor cortex. The exact cause of ALS has not been fully elucidated, however the accumulation of aggregates of misfolded proteins such as mutant superoxide dismutase 1, fused in sarcoma, ubiquilin 2, TAR DNA-binding protein 43, and peptides derived from intronic repeats of the *C9ORF72* gene are key pathological hallmarks (132–134). These protein aggregates disrupt cellular homeostasis, leading to mitochondrial dysfunction, characterized by distorted cristae and membranes in spinal motor neurons, and impaired calcium buffering capacity (135). Additionally, increased ROS production and altered adenosine triphosphate (ATP) metabolism contribute to cellular stress and apoptosis. Neuroinflammation is recognized as a critical component of ALS pathology. Microglial activation is evident in early stages, and as the disease progresses, damaged motor neurons release “danger signals” that polarize microglia into a pro-inflammatory M1 phenotype, characterized by increased secretion of cytokines such as IL-1β, IL-6, and TNFα. This inflammatory milieu likely contributes to the elevated CRP levels observed in ALS (136). The severity of ALS is commonly assessed using the ALS Functional Rating Scale-Revised (ALSFRS-R). Interestingly, several studies have reported a strong correlation between CRP levels and ALSFRS-R scores, suggesting a link between inflammation and disease progression, while other studies have yielded conflicting results. For instance, Keizman et al. found a strong association between high CRP levels and ALSFRS-R scores, indicating that CRP may be directly proportional to disease progression (137). Similarly, Ryberg et al. (2010) observed significantly elevated CRP levels in the cerebrospinal fluid of ALS patients compared to controls (138). However, Beers et al. reported inconsistent findings across two cohorts: in one cohort, higher CRP levels were associated with rapidly progressing ALS, while in the other, elevated CRP was observed in both fast and slow progressing ALS (139). Furthermore, another study found elevated CRP levels specifically in the cerebrospinal fluid and plasma of ALS patients with *C9ORF72* expansions, but not in the overall ALS group, suggesting potential differences in peripheral and central inflammatory regulation (140). To further investigate the relationship between CRP and ALS risk, a population-based study involving 289 ALS patients (65.7 ± 10.5 years of age) and 506 controls was conducted. ALS patients were categorized by smoking prevalence and body mass index. Although a mutually adjusted model showed no statistically significant association of ALS with CRP, a higher risk of ALS for the top quartile of CRP was observed when the model was adjusted for body mass index. These findings highlight the complex relationship between CRP and ALS, warranting further investigation to fully elucidate its role in disease pathogenesis and progression (141).

### 4.4 CRP and Huntington’s disease

Huntington’s disease (HD) is an autosomal dominant neurodegenerative disorder caused by CAG repeat expansions in exon-1 of the Huntingtin (HTT) gene, resulting in an elongated polyglutamine (polyQ) chain within the Huntingtin (Htt) protein. The mutant Htt (mHtt) protein accumulates predominately in the basal ganglia, cerebellum, and striatum, leading to motor control dysfunction (142). Unlike AD and PD, the genetic etiology of HD is well-established, with mHtt containing polyQ repeats exceeding 35 residues playing a central role in its pathogenesis (143). However, the precise mechanisms underlying neuronal death and neuroinflammation remain incompletely understood. Normal Htt plays a vital role in cellular homeostasis, interacting with cytosolic proteins and regulating apoptosis pathways (144). For instance, in the striatum, normal Htt protects cells from apoptotic stimuli, including Bcl-2 homologs (145). Additionally, Htt participates in other crucial neuronal processes, such as exocytosis, endocytosis, and synaptic vesicle trafficking (146, 147). The vulnerability to mHtt toxicity is not uniform across neuronal populations, with specific neuronal groups exhibiting heightened susceptibility. For example, the medial and dorsal striatum show greater degeneration compared to the lateral and ventral striatum. Recent studies have explored the role of mHtt in triggering immune responses and contributing to neurodegeneration. mHtt appears to activate microglia and astrocytes, inducing the secretion of pro-inflammatory cytokines such as IL-6 (148). Moreover, mHtt positively regulates the Nuclear factor-κB signaling pathway, leading to the release of IL-8 and IL-6 (149). Consistent with these findings, HD patients exhibit a distinct inflammatory profile, with increased levels of IL-4, IL-10, TNFα, and CRP observed as the disease progresses (150). However, meta-analyses examining the correlation between CRP levels and HD have yielded inconsistent results. For example, Sánchez-López et al. reported elevated CRP levels in later-stage HD, but lower levels in early-stage subjects compared to controls (151). Conversely, Wang and colleagues found significantly elevated CRP levels in premanifest HD compared to both healthy and familial controls, but no significant elevation in manifest HD (152). These conflicting findings suggest that the relationship between CRP levels and HD is complex and potentially influenced by disease stage. The observed increases in CRP could reflect an acute-phase response. A comprehensive, systematic study encompassing all stages of HD is crucial to elucidate the precise role of CRP as a biomarker in disease progression.

## 5 CRP and other amyloidosis diseases

Cardiac amyloidosis (CA), another proteinopathy of significant clinical interest, involves the extracellular deposition of insoluble amyloid fibrils within the myocardium, leading to infiltrative cardiomyopathy (153). Transthyretin (TTR), a plasma protein primarily synthesized in the liver and responsible for transporting thyroxine and retinol-binding protein, can misfold and form fibrils, resulting in transthyretin amyloid cardiomyopathy (ATTR-CA) (153). ATTR-CA is classified into two subtypes: hereditary ATTR amyloidosis (ATTR-h), characterized by mutations in the TTR gene and a broad range of clinical manifestations, and wild-type ATTR amyloidosis (ATTR-wt), where native TTR misfolds, predominantly affecting older individuals and causing heart failure (154, 155). Pathologically, these subtypes overlap, with the primary distinction being the presence of mutant versus wild-type TTR gene sequences. Fibrils in ATTR-CA can be further categorized based on the TTR length: ATTR type-A, containing a mixture of full-length and truncated TTR, and ATTR type-B, containing only full-length TTR (156). While comprehensive meta-analyses on the association between CRP levels and ATTR-CA are limited, studies have demonstrated a correlation. For instance, research investigating the link between cardiac global longitudinal strain and inflammatory markers in ATTR patients revealed a significant association between CRP and ATTR (157). Notably, CRP levels correlated strongly with the E/e’ ratio, a measure of diastolic function (r = 0.58, p < 0.05), and IL-6 levels correlated with right ventricular global longitudinal strain (r = 0.881, p < 0.05). Elevated IL-6 can upregulate CRP expression, potentially leading to the formation of pro-inflammatory CRP isoforms (mCRP and pCRP*), which may further impair cardiac function. A large study involving 2,566 ATTR patients found that 27% exhibited elevated CRP levels; however, while multiple inflammatory markers were associated with mortality, CRP was not identified as the sole determinant of disease severity (158). These findings suggest that while elevated CRP is common in ATTR-CA, its precise prognostic role requires further investigation. In addition to ATTR-CA, immunoglobulin light-chain (AL) amyloidosis also affects the myocardium, with 70–80% of systemic AL amyloidosis cases involving cardiac involvement (159–161). Unlike ATTR-CA, AL cardiomyopathy carries a poor prognosis, with median survival less than one year in patients with heart failure (162). AL amyloidosis is characterized by the proliferation of monoclonal immunoglobulin light chains from indolent B-cell clones, leading to amyloid accumulation in various organs, including the heart. Although AL amyloidosis is not traditionally considered an inflammatory disease like neurodegenerative amyloidosis, which involves immune cell activation and cytokine release, elevated levels of metalloproteinases (involved in extracellular matrix homeostasis) suggest an inflammatory milieu within the AL heart. A recent study involving 165 AL cardiomyopathy patients with increased relative myocardial wall thickness possessing higher CRP levels were associated with a higher risk of mortality (163). While these findings indicate that CRP may be a potential prognostic marker in AL cardiomyopathy, further validation in larger cohorts is needed to elucidate the mechanistic insights into a potential CRP-driven inflammatory response in this condition.

## 6 Conclusion and perspectives

To date, the exact mechanism of CRP dissociation on amyloid plaques remains unclear. While pCRP* and/or mCRP was found localized to Aβ plaques in AD brain tissue, the plaques have the capacity to dissociate pCRP *in vitro* (42), suggesting a reciprocal influence that may contribute to localized inflammation near plaques due to CRP activation (42). Consistent with CRP’s pro-inflammatory role, studies indicate that pCRP* and/or mCRP, not pCRP, co-localizes with Aβ plaques and initiates C1q-dependent cortical inflammation in AD (42). Despite recent studies suggesting a clinically relevant novel mechanism of CRP activation (30), the PCh-dependent interaction with activated cell membranes is still considered the primary mediator of pCRP dissociation (164) and likely plays a substantial role in pCRP* and/or mCRP formation in AD. Intriguingly, therapeutic targeting of CRP dissociation by blocking PCh interaction with a low molecular weight inhibitor has shown promise even for chronic conditions (22). While CRP lowering strategies like CRP apheresis are effective in acute settings (165), a low molecular weight inhibitor of CRP dissociation offers a potentially more convenient administration route for chronic conditions like amyloidosis (165). However, there is also evidence that the binding face (i.e., B‐face, **Figure 1**) of pCRP interacts with misfolded or aggregated proteins, i.e., proteins whose secondary structure is predominately β-sheets, by a mechanism independent of the PCh binding pocket (166, 167). Therefore, the exact pathophysiological role of CRP in amyloidosis and potential mechanisms of CRP activation remains an area of significant research interest. Nevertheless, the significant role of CRP in amyloidosis and especially AD is undoubted. Consistently, elevated CRP levels have been reported in AD patients (168) and linked to accelerated disease progression (169).

Despite the growing body of research highlighting the involvement of CRP in amyloid-based pathologies, several critical questions remain unanswered. First, the temporal relationship between protein aggregate formation and inflammation requires further elucidation. It remains unclear whether the accumulation of protein aggregates initiate inflammatory cascades or *vice versa*, i.e., pre-existing inflammation drives the formation and subsequent aggregation of misfolded proteins. Second, the precise nature of the interaction between CRP and protein aggregates warrants further investigation, since it is ambiguous whether CRP possesses a specific affinity for these aggregates, and if so, what is the precise nature of CRP-amyloid binding. Third, the mechanisms underlying CRP’s conformational changes in the presence of protein aggregates remain unclear; more precisely, how does the conformational transition of CRP from its pentameric to monomeric form occur in this context. And finally, given the inherent instability of mCRP and its propensity to aggregate (30), the potential for mCRP to act as a seeding agent in amyloidosis need to be addressed. Does mCRP exhibit seeding effects, contributing to the propagation of amyloid fibrils, and if so, is the inhibition of mCRP formation a relevant new target in proteinopathy therapy? Addressing these fundamental questions will be crucial for advancing our understanding of CRP’s role in protein misfolding diseases and ultimately for the development of targeted therapeutic interventions.
